# Delayed Development of Feeding Skills in Children with Feeding Difficulties—Cross-sectional Study in a Brazilian Reference Center

**DOI:** 10.3389/fped.2017.00229

**Published:** 2017-10-31

**Authors:** Cláudia C. Ramos, Priscila Maximino, Rachel H. V. Machado, Ana Beatriz Bozzini, Letícia W. Ribeiro, Mauro Fisberg

**Affiliations:** ^1^Instituto PENSI, Hospital Infantil Sabará, Fundação José Luiz Egydio Setúbal, Higienópolis, Sao Paulo, Brazil

**Keywords:** feeding difficulties, feeding skills, childhood, complementary feeding, feeding behavior

## Abstract

**Background:**

Delays in gross motor development, sensory processing issues, and organic and behavioral problems are known to interfere in the development of feeding skills (FS); and—therefore—in the success of the process of feeding a child. Children with feeding difficulties (FD) commonly present inadequacy of FS.

**Objectives:**

Assessment of five FS in Brazilian children with FD, and search of associations with types of FD.

**Methods:**

Cross-sectional study with 70 children below 10 years old. Data were obtained from medical records: age, gender, age at texture transitions, feeding phase (breastfeeding, weaning to solids or full solids) at first complaint; characteristics of the meal (duration, environment, and shared meals with adults), self-feeding practices, use of feeding equipment and bottle, mouthing, feeding position and FD diagnosis. Skills were categorized according to standards for age. Chi-Square, Anova Test (or non-parametric equivalent) and Multinomial logistic regression tests were used, with a significance level of 5%.

**Results:**

There was no difference in FS (*p* > 0.05) or in the number of FS inadequateness (*p* = 0.84) according to FD diagnosis. The majority (94%) of children presented at least one delayed development of FS; 1/3 presented delays in more than half of the FS. The most prevalent inadequacies in FS were inadequate feeding position (73.5%), prolonged bottle feeding (56.9%), and inadequate self-feeding practices (37.9%). Feeding complaints first appeared at 10.9 ± 11.4 months, and picky eating was the most prevalent type of FD (37.1%). Most children were fed in inadequate environments (55.2%), without the company of adults (78%). Transition to solid foods occurred at 16 ± 5.6 months. Multinomial logistic regression showed no difference in likelihood of presenting any type of FD compared to picky eating, according to FS. Age at texture transition both from breastfeeding to complementary feeding (*p* = 0.95), and from complementary feeding to solid foods (*p* = 0.43) did not vary according to FD diagnosis.

**Conclusion:**

FS development or number of FS inadequateness did not vary according to FD diagnosis. Identification of these inadequacies could help the discussion for multi-professional treatment of patients with FD.

## Introduction

It is well established that oral feeding processes depend on both motor skills and reflexes that work in synergy (1), and which maintain close relationship with brain development and its functions. Oral feeding skills (FS) have commonly been conceptualized as an infant’s ability to organize and coordinate oral-motor functions to efficiently achieve nutritional needs (2). The learning process depends on stimulation and individual variations, although patterns tend to be alike in most infants. In addition, sensory processing issues may also contribute to this individual variation (1, 3, 4).

Among other aspects, typical development of FS is described as physiologic maturity to gradually displace breast milk from 4 to 6 months of age (5–7), as well as developmental readiness for postural control and for introduction of complementary foods (4, 5); discriminative mouthing from 4 months of age on (which offers opportunities for sensorial perception and discrimination and stimulates biting and chewing movements; serving as preparation for future feeding experiences) (8, 9); progressive self-feeding practices as child grows (10); and use of cups in different shapes and sizes—besides other feeding equipment—from 12 months of age on (4, 11) [after 24 months old, prolonged use of bottles is both unnecessary (4) and prejudicial to typical development of swallowing mechanisms, occlusion and speech, aside from favoring inadequate feeding choices, which increase the risk for cavities and overweight (5)].

Delays in gross motor development, sensory processing issues, and organic and behavioral problems are known to interfere in the development of FS; and—therefore—in the success of the whole process of feeding a child (1, 3). Hence, one could hypothesize as to the relationships between altered FS and feeding difficulties (FD) often reported in early childhood, such as disruptive mealtime behavior, rigid food preferences and food refusal, picky eating, phobia, extreme agitation, among others. Kerzner et al. (12) group FDs into “children with limited appetite,” “agitated children,” “phobic children,” “misperception of caregivers,” “picky eating,” and “organic causes.”

Feeding difficulties are estimated as 25–45% prevalent in children with typical development, and up to 80% prevalent in children with health conditions and/or developmental disabilities (10, 13). Moreover, its implications can impact on behavioral, psychological, nutritional, and growth patterns, temporarily or persistently (8, 12). Causes of FD are multifactorial and include delays in motor skills development, impaired sensorial processing or inadequate feeding behaviors (8, 10, 12, 14). Empirical observation during follow-up of children with FD commonly report inadequacy of FS, which are described by health professionals as persistent and difficult to manage. These inadequacies do not present themselves as a uniform pattern, and—among the different types of FD mentioned earlier—it is often clinically reported that children with organic diseases who have feeding complaints tend to specifically reject feeding equipment and new textures; while picky eaters have no difficulty with these factors, but may present difficulties with texture transitions and prefer milk over food, tending to prolonged bottle feeding. In this context, health professionals may hypothesize that these group of children do present altered patterns of FS when compared to reference scales (4–7, 11, 15), and that these inadequacies may vary according to the type of FD.

Thus, seeking answers to such hypothesis may allow both early identification of risk factors and development of diagnostics methods and multidisciplinary treatment of these complaints. The objective of this study, therefore, is to characterize the profile of five FS (bottle feeding, self-feeding practices, proper use of feeding equipment, proper feeding position, and mouthing) in a group of Brazilian children with different types of FD, as well as to seek possible associations with the FD diagnosis.

## Materials and Methods

### Study Design and Sampling

It is a cross-sectional study, carried out in the Center of FD—an outpatient service dedicated to support children and teenagers between 0 and 18 years old, with complaints of FD [excluding cases of psychiatric eating disorders diagnosed according to DSM-5 (16)]. The center is part of Instituto PENSI/Hospital Infantil Sabará/Fundação José Luiz Egydio Setúbal, located in São Paulo, Brazil. Sampling was assembled by convenience, with the inclusion of all patients up to 10 years old followed by the service until to the moment of the data collection (August/2014 to Feburary/2016; *n* = 70; total population *n* = 77). The adolescents were excluded from sampling (*n* = 7) due to the full maturity of FS, which could interfere with interpretation of results. All patients presented written consent forms signed by their responsible caregiver, after ethical approval of the project (CAAE 32939314.0.0000.5567; approval granted in 13/08/2014 under document n. 808.394).

### Data Collection

Data were collected from the interviews with caregivers, as part of the service protocol. The protocol consists of a joint appointment with a pediatrician, speech therapist and nutritionist, followed by a multidisciplinary discussion of each case. Each child is diagnosed as to the type of FD as criteria suggested by Kerzner et al. (12), as mentioned above. Families receive then a feedback, with indication of a therapeutic plan designed by each specialty, such as diet plans and nutritional education activities, medication, stimulation, and reestablishment of oral functions or even referral to other professionals from other areas. Several subsequent follow up appointments may be scheduled after this, as suggested by the multidisciplinary team. Nevertheless, data were always collected at the initial appointment at the Center. The guidelines used by each specialty were defined according to standards for age, and are described both by Maximino et al. (17) and further below.

The collected information from these appointments was then reported in the child’s medical records, from which the following variables were selected: age (months), gender, age (months), and feeding phase (breastfeeding, weaning to solids or full solids) at first feeding complaint; and characteristics of the meal (duration in minutes, meal environment, and shared meals with adults). Meal environment was deemed adequate if provided opportunities for proper posture to eat and for children’s self-feeding practices (which require a table and chairs), as well as the opportunity to share meals with members of family and learn about feeding behaviors; hence compatible with kitchen and dining room environments (18). Preterm children currently under 24 months old were age-adjusted for all parameters evaluated.

To assess FS, age (months) at texture transitions (introduction of complementary foods and weaning to solids), self-feeding practices, use of proper feeding equipment (sippy cups, bottles, and spoon), presence of mouthing (retroactive data for children over 12 months of age), and feeding positions at meals were collected. Data on age at texture transition and at use of bottles has also been adjusted for preterm children, regardless of their age in the moment of data collection. FD diagnosis was made by the multidisciplinary team, in agreement with the criteria suggested by Kerzner et al. (12). FS were categorized according to standards of normality described elsewhere (4–7, 11, 15), all summarized below:

- Texture transitions from breastfeeding to complementary foods is expected at 6 months, and weaning to solid foods at 12 months of age;
- Initial use of glasses and sippy cups are expected at 6 months, gaining full maturity at 24 months of age. Self-feeding through finger foods and use of straw are expected at 9 months, and self-feeding through spoon at 18 months of age;
- Discriminative mouthing is expected from 4–5 months old on;
- Feeding position at meals are categorized according to age:

○ Up to 6 months: varied feeding positions that provide physical comfort to both caregiver and child during breastfeeding and/or bottle feeding.
○ 6–7 months on: use of high chair or special chair seat back at a 90° inclination.
○ 18–24 months on: use of high chair, infant chair/table, or a booster seat placed on an adult chair at the table.

### Statistical Analysis

Consistency of data was evaluated, and statistical analysis was performed by SPSS v21 software. Descriptive analysis was conducted through frequency of distribution (%), average ± SD, and quartiles p25%, p75%. Chi-Square, Anova Test (or non-parametric equivalent) and Multinomial logistic regression tests were used to check for associations between FS and FD diagnosis, with a 5% significance level.

## Results

General characteristics of population and mealtimes are described in Table 1 according to age group. It is noteworthy that the greater the age group, the later feeding complaints started. Children followed at the center below 12 months of age started with complaints during breastfeeding/formula feeding periods; children between 1 and 6 years old started complaints mostly during complementary feeding; while older children (more than 6 years old) started complaints around 3 years old. The most common type of FD among children below 24 months of age was Misinterpretation of caregivers; while the most prevalent type among older children was picky eating.

**Table 1:** General characteristics of children according to age group.

	% or Mean ± SD (*N*)				
	0–12 months	13–24 months	2–6 years	6–10 years	Total
**Gender**					
Female	36.4% (*n* = 4)	64.3% (*n* = 9)	21.1% (*n* = 8)	28.6% (*n* = 2)	32.9% (*n* = 23)
Male	63.6% (*n* = 7)	35.7% (*n* = 5)	78.9% (*n* = 30)	71.4% (*n* = 5)	67.1% (*n* = 47)
**Age (adjusted for prematurity)**	8.73 ± 2.5 months (*N* = 11)	18.3 ± 3.6 months (*N* = 14)	3.3 ± 1 years (*N* = 38)	7.7 ± 1.4 years (*N* = 7)	3 ± 2.1 years (*N* = 70)
**Age at first complaint**	3.1 ± 2.9 months (*N* = 11)	7.2 ± 3.8 months (*N* = 14)	10.5 ± 6.8 months (*N* = 38)	2.8 ± 1.8 years (*N* = 7)	10.9 ± 11.4 months (*N* = 70)
**Feeding phase at first complaint**					
Breastfeeding	54.5% (*n* = 6)	14.3% (*n* = 2)	17.9% (*n* = 7)	0% (*n* = 0)	21.4% (*n* = 15)
Complementary feeding	45.5% (*n* = 5)	71.4% (*n* = 10)	28.2% (*n* = 11)	16.7% (*n* = 1)	38.6% (*n* = 27)
Solid foods	0% (*n* = 0)	14.3% (*n* = 2)	53.8% (*n* = 21)	83.3% (*n* = 5)	40% (*n* = 28)
**Duration of exclusive breastfeeding (months)**	2 ± 2.4 months (*N* = 11)	2.1 ± 2.3 months (*N* = 14)	3.2 ± 2.9 months (*N* = 36)	2.7 ± 2.5 months (*N* = 6)	2.7 ± 2.7 months (*N* = 67)
**Age (months) at texture progressions**					
Introduction to complementary foods	5.2 ± 0.8 months (*N* = 5)	5.6 ± 0.9 months (*N* = 11)	5.3 ± 0.9 months (*N* = 27)	3.8 ± 1.7 months (*N* = 4)	5.2 ± 1.1 months (*N* = 47)
Weaning to solids	–	15.8 ± 4.4 months (*N* = 4)	15.7 ± 5.8 months (*N* = 19)	24 months (*N* = 1)	16 ± 5.6 months (*N* = 24)
**Feeding difficulties diagnoses**					
Agitated	–	14.3% (*N* = 2)	5.1% (*N* = 2)	–	5.6% (*N* = 4)
Limited appetite	18.2% (*N* = 2)	7.1% (*N* = 1)	25.6% (*N* = 10)	–	18.3% (*N* = 13)
Phobia	9.1% (*N* = 1)	7.1% (*N* = 1)	5.1% (*N* = 2)	–	5.6% (*N* = 4)
Misinterpretation	45.5% (*N* = 5)	42.9% (*N* = 6)	7.9% (*N* = 3)	14.3% (*N* = 1)	21.4% (*N* = 15)
Organic	27.3% (*N* = 3)	7.1% (*N* = 1)	7.9% (*N* = 3)	14.3% (*N* = 1)	11.4% (*N* = 8)
Picky eating	–	21.4% (*N* = 3)	47.4% (*N* = 18)	71.4% (*N* = 5)	37.1% (*N* = 26)
**Meal environment**					
Adequate	50% (*N* = 4)	20% (*N* = 2)	47.1% (*N* = 16)	66.7% (*N* = 4)	44.8% (*N* = 26)
Inadequate	50% (*N* = 4)	80% (*N* = 8)	52.9% (*N* = 18)	33.3% (*N* = 2)	55.2% (*N* = 32)
**Shared meals**					
Presence	9.1% (*N* = 1)	36.4% (*N* = 4)	18.2% (*N* = 6)	50% (*N* = 2)	22% (*N* = 13)
Absence	90.9% (*N* = 10)	63.6% (*N* = 7)	81.8% (*N* = 27)	50% (*N* = 2)	78% (*N* = 46)
**Duration of meals (min)**	65 ± 35.4 (*N* = 2)	58.8 ± 41.2 (*N* = 8)	39.5 ± 20.5 (*N* = 10)	42.5 ± 10.6 (*N* = 2)	49 ± 30.3 (*N* = 22)

*Center of Feeding Difficulties. Instituto PENSI, 2016*.

**Figure 1:**
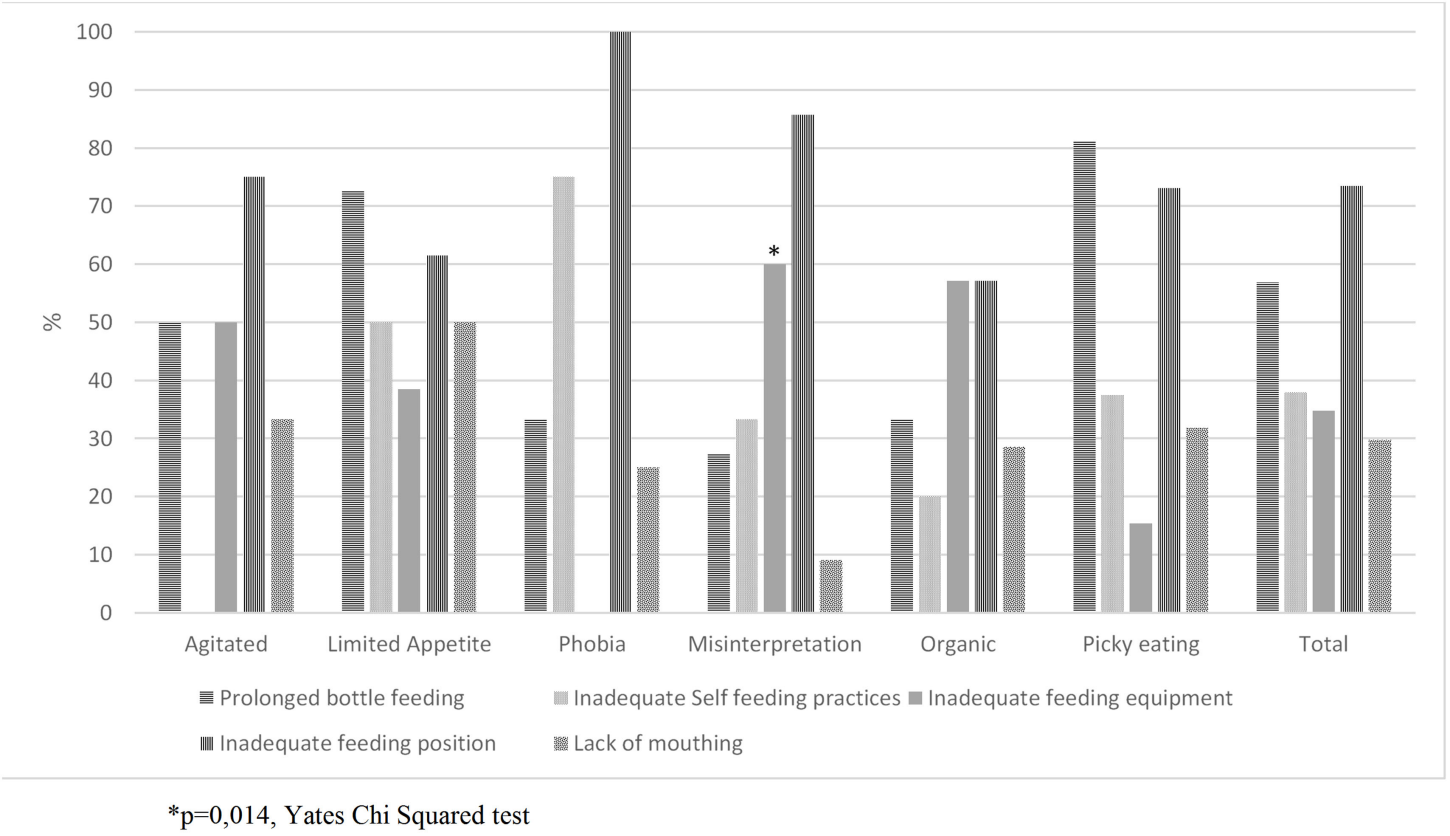
Prevalence of inadequate feeding skills according to types of feeding difficulties.

In Figure 1, prevalence of inadequate FS was compared to FD diagnosis. Only the use of feeding equipment was associated to different types of FD: children who were diagnosed as misinterpretation of parents were related to inadequate use of feeding equipment (*p* = 0.014). The four remaining FS were not associated to types of FD (*p* > 0.05). The most prevalent FS inadequacies were “inadequate feeding position” (73.5%), “prolonged bottle feeding” (56.9%) and “inadequate self-feeding practices” (37.9%). According to Multinomial logistic regression (Table 2), picky eating did not differ from all other typed of FD combined regarding any of the FS analyzed.

**Table 2:** Multinomial logistic regression results.

Feeding difficulties type		B	Sig	Exp (B)	CI 95%	
All remaining types of FD combined	Prolonged bottle feeding	−1.9	0.059	0.146	0.020	1.07
	Lack of self-feeding practices	0.73	0.42	2.1	0.35	12.43
	Inadequate feeding equipment	−0.93	0.30	0.39	0.067	2.31
	Inadequate feeding position	−0.41	0.64	0.66	0.12	3.78
	Lack of mouthing	−0.75	0.44	0.48	0.072	3.13

*Reference category = Picky eating*.

*Instituto PENSI: 2016*.

FS were also categorized as to the number of altered skills presented. Children presented—in average—two altered FS ± 1.05 (varying from 0 to 5 alterations). Only about 5.8% (*n* = 4) children presented all normal FS, around 60.9% (*N* = 42) presented 1 to 2 alterations, and the remaining 33.3% (*n* = 23) presented three or more altered FS. The ANOVA test showed no significant association between number of alterations and FD diagnosis (*p* = 0.84). Age at texture transition both from breastfeeding to complementary feeding (*p* = 0.95; ANOVA test), and from complementary feeding to solid foods (*p* = 0.43; Kruskal–Wallis test) did not vary according to FD diagnosis as well.

## Discussion

Data presented in this study showed no significant difference in FS or in the number of FS inadequateness conforming to FD diagnosis. Nevertheless, the majority (94%) of the sample presented at least one delayed development regarding FS, and 1/3 of children assessed presented delayed development in more than half of the FS evaluated. Overall, feeding complaints first appeared in the first year of life (feeding phase compatible with complementary feeding and transition to solid foods), and the most prevalent FD was picky eating. Most children were fed in inadequate environments and without the company of adults; did not self-feed, and were usually fed in inappropriate feeding positions. Most children also practiced prolonged bottle feeding; and transition to solid foods occurred at later stages when compared to reference. Few studies have been found to discuss such skills in children with FD, specifically. However, there are data on the development of such skills in healthy children in the same age group, which will be discussed further along, conforming to each type of skill.

### Prolonged Bottle Feeding

In Brazil, the “bottle culture” is widely diffused and accepted by families, despite the recommendations for its gradual replacement between 12 and 24 months of age (7). In a Brazilian study with 502 children up to 6 years old (19), around 32% of prolonged bottle feeding was identified from 24 to 47 months of age. Present results were even higher, since prolonged bottle feeding was found to be 56.9% prevalent in children between 2 and 6 years old. In contrast, a study conducted in an American outpatient service with 126 children (20) reported prolonged bottle feeding in 4% of 24-month-old children. The difference between Brazilian and American data highlights that socioeconomic and cultural factors can be associated to nutritive and non-nutritive sucking (19). Moreover, bottle feeding is frequently associated to milk intake, and prevalence of its prolonged use is associated to caregivers’ concern regarding decreases in consumption of milk (21).

This situation suggests that the profile described herein could be either characteristic of the Brazilian population or a reflection of feeding complaints. Although results herein did not show association of prolonged bottle feeding and types of FD, this may only mean that all types of diagnosis may lead to the same outcome (drinking more milk). Moreover, around 81% of children diagnosed as picky eaters, and 72% of those classified as with limited appetite presented prolonged bottle feeding; matching the clinical concern with this practice—milk becoming a replacement for meals, which may decrease appetite and favor inadequate feeding choices.

A study conducted with 455 parents of 30-month-old children (22)—in which 20% prevalence of FD was detected—showed that the excessive consumption of milk was related to low appetite during meals; while Maximino et al. (17) have also mentioned the tendency for an increase in the ingestion of proteins from milk/formula in a descriptive study of Brazilian children with FD. Kerzner et al. (12) considered prolonged bottle feeding a suggestive symptom of FD; and Noppornlertwong et al. (23) also mentioned a relation between children with adequate feeding patterns, lower use of bottle at 15 months of age, better acceptance of foods and lower prevalence of picky eating. The primordiality to advice on the discontinuance of prolonged bottle feeding is clear regardless of the FD diagnosis, in order to preserve normal development of swallowing, occlusion or speech, and to prevent inadequate feeding substitutions.

### Self-Feeding Practices

Self-feeding is an important step toward the development of independence and self-confidence, besides interfering in quality of chewing and swallowing, and all sensory and emotional aspects involved in feeding, as explained previously (5, 11, 24). Carruth et al. (25) stated that children develop skills to self-feed in the first 2 years of life. Those who develop this skill between 7 and 14 months of age tend to present higher intake of nutrients, and the majority already self-fed or demonstrated skills for such between 15 and 18 months of age. In the current sample, however, most children (97%) did not self-feed as reported by parents, even the older children assessed. In a study conducted with 349 children with FD, from 1 month to 12 years of age, deficits in self-feeding were also reported in most participants (26). The American Academy of Pediatrics suggests that both capacity of sitting and mouthing are denotative of the beginning of the process of self-feeding through finger foods (7); and lack of autonomy is considered by Kerzner et al. (12) as a symptom suggestive of FD. The continuance of this behavior is considered an impediment for better progress and treatment.

In the present sample, all children diagnosed as agitated did self-feed, opposed to other types of FD assessed (*p* > 0.05). These children are usually reported by literature as those who first develop feeding complaints during the transition to self-feeding, refusing to remain seated during meals, eating small amounts of food, and frequently failing to gain weight (12); hence, developing ineffective self-feeding patterns. This remarks the importance of evaluating both presence and patterns of self-feeding practices in children with FD. On the counterpart, phobic children were the group who least self-fed in our sample (25%) and moreover did not use proper feeding equipment and were not fed in adequate postures. Such behaviors are expected within typical characteristics of phobia since these children lack experience, and/or feel threatened when food is introduced orally, having possibly lost feeding milestones or been forcefully fed by caregivers (12).

### Feeding Equipment and Positions

Feeding equipment should match the child’s developmental status through the toddler period, and, as stated by Morris and Klein (4), children with feeding delays and difficulties are typically older when they are introduced to most feeding and oral-motor equipment. Since the development of motor skills is secondary to children’s experiences with their environment, children who experience problems with postural tone and movement may experience added challenges in learning to eat with proper feeding equipment. A good body position will make it easier for the baby to relax and use good mouth control (4). van den Engel-Hoek et al. (27) stated that the learning process toward the correct use of feeding equipment in children younger than age 8 months can take from 2 to 10 weeks, regardless of the age in the beginning of the practice, and that inadequate behavior during meals was reduced proportionally to the development of the handling of feeding equipment. Given that 1/3 of the assessed sample presented inadequacies in feeding equipment use, it is important to consider the evaluation of feeding equipment in cases of FD.

In the present sample, inadequateness of feeding positions were reported in 73.5%. National data (5) showed similar results: at 12 months of age, only 24% of children studied were fed on a high chair in the study group, and 19% in control group. Several feeding positions were identified, such as in the stroller, on the lap, on the couch, in the baby walker, sitting on the table or on inappropriate chairs. This information points out toward a general inadequacy among Brazilian children, and a similar profile is frequently observed during clinical follow up of children with FD. Phobic children and misperceived groups were the ones who most lacked FS related to feeding position and equipment in the present sample (*p* > 0.05).

To our knowledge, few studies have addressed the association between feeding problems and feeding positions. One of them has found that Thai children with feeding problems (42.6%) had no definite feeding site more often than children without feeding complaints (31%). These children were usually fed at appropriate sites, such as in a high chair or at the child’s table or at the family table more frequently than feeding-problem children. Feeding-problem children were fed less frequently, were less likely to be fed at their own table or at the family table, and had meal durations longer than 30 min, compared to normal children, mostly due to routine adjustment provided by caregivers pursuant to where the child would accept to eat; highlighting the need to educate parents regarding such behaviors (28).

### Mouthing

As previously mentioned, complementary feeding should be guided, among other factors, by readiness for varied textures of foods (which is enhanced by mouthing); and infants who lack mouthing due to sensory problems are bound not to have a good oral sensorimotor skills development, leading possibly to food refusal or food aversion (4, 9, 29, 30). In a case–control study with children presenting oral tactile defensiveness (which can affect patterns of mouthing) (31), habits and feeding choices differed in study and control groups, with characteristics very similar to those observed in FD. Children presented a tendency toward low appetite, hesitation to try new foods, refusal to eat in other people’s homes, and refusal to certain foods according to smell and temperature. They also presented difficulties in eating vegetables, got frequently nauseated and/or bit their lips and cheeks, presented aversion to textures, and consistencies of food, when compared to control group. Similarly, Farrow and Coulthard (14) described an association between picky eating and sensorial processing, especially tactile, olfactory, and gustatory.

In the current sample, 29.8% of children presented restricted sensorial exploration patterns through mouthing. Surprisingly, the highest prevalence of inadequacy in mouthing was observed in children with limited appetite (50%). However, no further studies were found to add to the discussion of these variables. Despite lack of association between this skill and types of FD, picky eaters frequently make food choices that follow specific patterns according to the sensory characteristics of foods (32), and the altered pattern of mouthing is usually observed during speech therapy sessions (9), providing indications that there may be, in fact, a relation between both.

### Texture Transitions

Results here described regarding texture transitions are similar to those of de Macedo (5), who studied Brazilian children of up to 12 months of age—without specific diagnosis of FD—and described the introduction of puréed foods at 5.4 and 5.5 months old in the study and control groups, respectively; and weaning to solids at 7.8 months old in the study group, and 9.6 months in the control group; while only 6.3% still had not received any solid foods by 12 months of age. Children from the current sample weaned to solids later along (an average of 16 months old) when compared to this study. Other studies conducted with healthy children with up to 24 months of age (15) reported acceptance of lumpy foods at around 8.7 months old and of solid foods without gagging or choking from up to 12.1 months of age. The official recommendation from Brazilian Health Ministry (33) states that introduction of complementary feeding should take place at 6 months of age, and weaning to solid foods at 12 months of age. According to these standards, in addition to the protocol adopted by the service, patterns of weaning to solid foods described herein are to be considered delayed.

Age at introduction to complementary feeding found in the current sample (5.2 months) is described by Hollis et al. (34) as an age group in risk of developing FD: children who have transitioned to complementary feeding at 6 months present a lower risk for FD than those between 4 and 6 months of age. Additionally, it is noteworthy that age range when feeding complaints first started were similar to age at texture transitions (5.2 months for complementary feeding, and 16 months for solids).

Although the type of FD was not associated with transition to other types of consistency and textures in this sample, it is known that this progression is one of the many important milestones in the development of infants and toddlers (11). Hence, could the complaint of FD be—in fact—the reflection or aggravation of a difficulty with texture processing? Guidelines from ESPGHAN (35) and the American Academy of Pediatrics (7) highlight the importance for health professionals to consider the signs of readiness for the beginning of texture transitions as a stage of children’s development. The role of professionals would be to guide parents to recognize this readiness and minimize the effects of the main difficulties.

Difficulties with textures could be related to delays in oral-motor development, sensory processing, organic and behavioral issues. These, in turn, could be related to FD. Moreover, the evaluation of type and texture of foods can be used to describe the relation between the acceptance and/or food refusal in children with FD (24). Taylor et al. (36) described children diagnosed as picky eaters in later childhood with reports of alterations in sucking patterns between 2 and 4 weeks of age; and state that the development of FD can be affected by the late introduction of solid foods. A study in the oral sensorimotor area and gross development (37) revealed that 31.8% of children had difficulty with the introduction of new foods, and a higher frequency of alterations in the gross motor and oral sensorimotor development was found in children who presented difficulties during weaning to solids, which may suggest a possible relation between this patterns and neuro-motor maturity (4, 5, 11). Additionally, children with oral sensorimotor issues may not process textures, volume, quantity, and temperature of the food in their mouths correctly, which can impact on the weaning process (1).

### Other Considerations: Parental/Caregiver Behavior

Eating is the first shared task in the relationship between caregiver and child; whereas any interference in this relationship can affect the style of interaction and the success of the feeding process (11, 13). The attitude of caregivers toward the feeding process can influence children’s autonomy by limited offer of learning opportunities (6). Moreover, the delay in oral-motor development can precisely lead to different interactions at mealtimes, with caregivers who are less susceptible or able to talk to the children and do not respond to physical signals of rejection. In addition, coercive practices contribute to the maintenance of immature patterns of FS; suggesting that interactions between caregivers and children while eating should be carefully observed (38, 39). Previous research has already concluded that interaction between parents and children is an important characteristic to be observed in cases of picky eating (7, 8, 13, 40, 41). Hence, strategies to improve FS in children with FD should also include monitoring of caregivers’ behaviors (28). Besides these factors, children in the present sample do not eat in company of adults (78%), which leads to questioning if whether the refusal reported is more influenced by undeveloped FS and/or other predisposition factors previously mentioned, or by lack of stimuli from home environment.

The study has its limitations, such as the restricted sample size and the absence of a control group (due to the nature of the reference center, focused on children with feeding complaints). On its strengths, current results offer an initial and relevant contribution around the topic, creating opportunities to deepen in the theme in further research.

## Conclusion

Children in the assessed sample were predominantly picky eaters, and presented at least one delayed development of FS. The most prevalent inadequacies in FS were inadequate feeding position, prolonged bottle feeding and inadequate self-feeding practices. Overall, feeding complaints first appeared in the first year of life. Weaning to solid foods was stimulated at later ages compared to official recommendations. There was no significant difference in FS or in the number of FS inadequateness according to FD diagnosis. Identification of these inadequacies could help the discussion for multi-professional treatment of patients with FD.

## Ethics Statement

This study was conducted according to the guidelines laid down in the Declaration of Helsinki and all procedures involving human subjects/patients were approved by the Ethical Committee (Instituto PENSI, Brazil), (CAAE 32939314.0.0000.5567; approval granted in 13/08/2014 under document n. 808.394). Written informed consent was obtained from all patients’ parents or legal guardians.

## Author Contributions

CR: main researcher, carried out the study and participated in the design, data analysis, and preparation on manuscript. RM: carried out the study and participated in the design, data analysis, and preparation on manuscript. AB, LR, PM: assisted in carrying out the study and preparation on manuscript. MF: supervised and assisted all phases of the study (Project PI).

## Conflict of Interest Statement

The PI of the project (MF) conferences in events, such as Abbott, CPW, EMS, Danone, Nestlé, Nutrociencia, PICME, Sanofi, Wyeth; scientific board member of Danone Institute International, Danone Research, Mondelez. Supports research projects at Abbott, CNPq, Coca-Cola, CPW, Danone Institute International, Danone Research, Fapesp, Fap Unifesp, Nestlé. PM consults for Hyproca Nutrition Nutrição Infantil Ltda. Authors have no participation in food, nutrition, or pharmaceutical companies, and there is no influence of any company in any of the projects, conferences, or publications conducted. The reviewer LM and handling editor declared their shared affiliation.
